# Ultraviolet B-induced oxidative damage in human skin keratinocytes is alleviated by *Pinus morrisonicola* leaf essential oil through activation of the Nrf2-dependent antioxidant defense system

**DOI:** 10.1080/13510002.2025.2527427

**Published:** 2025-07-02

**Authors:** Wan-Teng Lin, Yi-Ju Chen, Hsin-Ning Kuo, Suresh Kumar, Mosleh Mohammad Abomughaid, K. J. Senthil Kumar

**Affiliations:** a Department of Hospitality Management, College of Agriculture and Health, Tunghai University, Taichung, Taiwan; b Department of Surgery, Taichung Veterans General Hospital, Taichung, Taiwan; c Department of Animal Science and Biotechnology, College of Agriculture and Health, Tunghai University, Taichung, Taiwan; d Faculty of Health and Life Sciences, Management and Science University, Shah Alam, Malaysia; e Department of Medical Laboratory Sciences, College of Applied Medical Sciences, University of Bisha, Bisha, Saudi Arabia; f Center for General Education, National Chung Hsing University, Taichung, Taiwan; g Department of Biotechnology, Saveetha Institute of Medical and Technical Sciences, Saveetha University, Chennai, India

**Keywords:** *Pinus morrisonicola*, essential oil, ultraviolet B, photodamage, photoaging, HaCaT, antioxidant, Nrf2 pathway

## Abstract

**Background:**

Ultraviolet B (UVB) radiation contributes to skin disorders such as photodamage, photoaging, and cancer. Natural antioxidants can mitigate UVB-induced damage. *Pinus morrisonicola* (Taiwan white pine), known for its anti-cancer, anti-inflammatory, and antioxidant properties, is used in health-promoting beverages, but its skin-protective effects remain underexplored.

**Purpose:**

This study investigates the protective effects of *P. morrisonicola* leaf essential oil (PMLEO) against UVB-induced damage in HaCaT keratinocytes.

**Methods:**

HaCaT cells were exposed to UVB and treated with PMLEO. Cell viability, reactive oxygen species (ROS) levels, and antioxidant enzyme expression were assessed. The role of Nrf2, a key antioxidant regulator, was evaluated through knockdown experiments. The effects on UVB-induced melanogenesis were examined *via* α-MSH secretion followed by p53-mediated POMC expression.

**Results:**

PMLEO and *P. morrisonicola* bark essential oil (PMBEO) were non-cytotoxic up to 200 µg/mL. UVB reduced cell viability to 43%, but PMLEO co-treatment significantly restored viability and reduced ROS levels *via* Nrf2 activation, increasing NQO-1 and HO-1. Nrf2 knockdown impaired PMLEO's protection. PMLEO also inhibited UVB-induced α-MSH secretion by downregulating p53-mediated POMC expression, suggesting an anti-melanogenic effect.

**Conclusion:**

PMLEO protects dermal keratinocytes against UVB-induced oxidative stress, cell death, and melanogenesis via Nrf2 activation, highlighting its potential as a natural skin protectant.

## Introduction

Ultraviolet (UV) radiation, a major component of solar radiation, is a well-known cause of skin disorders, including actinic keratosis, photoaging, and skin cancer. UV-induced damage primarily results from oxidative stress, where an overproduction of reactive oxygen species (ROS) overwhelms the skin's antioxidant defenses. UVB (280–320 nm) and UVA (320–400 nm) exposure generate ROS, such as superoxide anions, hydrogen peroxide, and hydroxyl radicals, leading to oxidative damage of lipids, proteins, and DNA [1–3]. DNA lesions that escape repair contribute to carcinogenesis [4,5]. Effective preventive strategies, including dietary antioxidants and phytochemicals, can bolster the skin's defense against oxidative damage.

The nuclear factor erythroid 2-related factor 2 (Nrf2) signaling pathway plays a crucial role in cellular adaptation to oxidative stress. Under normal conditions, Nrf2 is bound to Kelch-like ECH-associated protein 1 (Keap1) in the cytoplasm. Under oxidative stress, Nrf2 dissociates from Keap1, translocates to the nucleus, and activates antioxidant response elements (AREs) in target genes [5]. This upregulates cytoprotective enzymes, including heme oxygenase-1 (HO-1), NAD(P)H:quinone oxidoreductase-1 (NQO-1), glutathione reductase (GR), glutathione S-transferases (GSTs), and superoxide dismutase (SOD), which mitigate ROS levels and repair oxidative damage. The Nrf2 pathway is a promising therapeutic target for oxidative stress-related skin conditions [5].

UVB radiation also stimulates α-MSH synthesis via p53-mediated pro-opiomelanocortin (POMC) gene regulation. UVB-induced DNA damage activates p53, leading to increased POMC expression and subsequent melanin production in melanocytes [6]. This provides photoprotection by absorbing UV radiation, but excessive melanin synthesis contributes to hyperpigmentation disorders such as melasma [7]. Oxidative stress enhances p53 activity while triggering other pathways, including p38 MAPK and JNK, further promoting melanogenesis [8]. The interplay between ROS and p53 in α-MSH production is critical in UVB-induced pigmentation responses [9].

Phytochemicals modulate Nrf2 signaling and other antioxidant pathways to protect against UV damage [10]. Upon UV exposure, phytochemicals facilitate Nrf2 nuclear translocation, enhancing HO-1, NQO-1, and SOD expression. These enzymes reduce oxidative stress, preventing DNA damage, inflammation, photoaging, and carcinogenesis [11,12]. Resveratrol, epigallocatechin gallate (EGCG), and curcumin, along with essential oils such as lavender, rosemary, and tea tree, inhibit UVB-induced α-MSH synthesis in keratinocytes [13]. These compounds reduce ROS levels, suppress p53 activation, and downregulate POMC expression, thereby mitigating UV-induced melanogenesis. Resveratrol and EGCG target oxidative stress pathways, while curcumin modulates NF-κB and p53 signaling to prevent α-MSH production [14]. Additionally, essential oils have been incorporated into sunscreens and cosmetics, increasing sun protection factor (SPF) values.

Over a thousand years, Asians have extensively utilized different parts of the pine tree, such as its pollen, cones, cortices, needles, and bark, for medicinal purposes and as food [15]. Particularly in China, Korea, and Taiwan pine needles, infused tea has been utilized as functional beverages. Consuming beverages made from pine needles can help treat various ailments, support hair growth, and enhance longevity [15]. Taiwan white pine (*Pinus morrisonicola* Hayata), a species native to Taiwan, is part of the Pinaceae family. Extracts of *P. morrisonicola* displayed a variety of pharmacological activities, including anti-oxidant [16–18], anti-inflammation [18,19], anti-cancer [20], and vasorelaxant [21] properties. A recent study reported that *P. morrisonicola* needle essential oil was extracted by the supercritical fluid extraction (SFE) method. A couple of SFE fractions exhibited strong antioxidative properties by reducing lipid peroxidation and preventing the formation of macrophage foam cells [16]. The ethyl acetate extract of fermented pine needles from *P. morrisonicola* (EAE-FPN) exhibits strong antioxidant activity, demonstrated by significant free radical scavenging effects against DPPH and hydrogen peroxide (H_2_O_2_), as well as enhanced antioxidant capacity measured by TEAC, attributable to its high phenolic and flavonoid content [19]. This antioxidant potential is particularly relevant since oxidative stress exacerbates skin conditions such as acne and rosacea by increasing reactive oxygen species (ROS), and reducing ROS may mitigate skin damage. Additionally, compounds isolated from the ethyl acetate fraction, including flavonoids and diterpenoids, inhibit matrix metalloproteinase-2 (MMP-2), an enzyme that degrades collagen and contributes to photoaging and impaired wound healing. Although purified compounds showed weaker inhibition of pro-MMP-2/9 compared to the crude extract, synergistic interactions within the extract likely enhance its overall efficacy in protecting skin collagen and delaying aging processes [19]. However, other bioactivities of *P. morrisonicola* essential oils were poorly studied. Thus, this study sought to examine the impact of *P. morrisonicola* essential oils on UVB-induced oxidative stress in human skin keratinocytes *in vitro*.

## Materials and methods

### Extraction of essential oils from the leaves and bark of *P. morrisonicola*

In March 2022, leaves and bark from *P. morrisonicola* were gathered at Guguan Hot Spring Park in Taichung, Taiwan. A total of 250 g of fresh leaves and 500 g of freshly peeled bark were placed in a 2-l flask with 1.5 l of distilled water. Hydrodistillation was conducted for 8 h using a Clevenger-type apparatus. After the extraction process, the essential oil was collected. The yield of the oil was calculated using the formula: Yield (%) = (weight of essential oil obtained/weight of air-dried plant material) × 100. The resulting leaf essential oil (PMLEO) and bark essential oil (PMBEO) were stored in airtight vials for later bioactivity testing.

### Chemicals and reagents

Dulbecco's Modified Eagle's Medium (DMEM), along with fetal bovine serum (FBS), penicillin, and streptomycin, were procured from Life Technologies located in Grand Island, NY. The 3-(4,5-Dimethylthiazol-2-yl)-2,5-diphenyltetrazolium bromide (MTT) assay reagent, 4′,6-diamidino-2-phenylindole (DAPI), and dichlorodihydrofluorescein diacetate (DCFH_2_-DA) were sourced from Sigma-Aldrich, St. Louis, MO. The antibody for γ-glutamate-cysteine ligase, catalytic subunit (γ-GCLC) was purchased from Cell Signaling Technology, Danvers, MA, while the glyceraldehyde 3-phosphate dehydrogenase (GAPDH) antibody was obtained from Santa Cruz Biotechnology, Inc. Antibodies for hemeoxygenase-1 (HO-1), NAD(P)H dehydrogenase quinone 1 (NQO-1), and Nuclear factor erythroid 2-related factor 2 (Nrf2) were supplied by Abcam, Cambridge, UK. Other chemicals used in the experiments were of analytical grade and were provided by Sigma-Aldrich or Merck, Darmstadt, Germany.

### Cultivation of cells and assessment of cell viability

HaCaT cell culture and treatment were conducted as described in our previous study [22]. Briefly, HaCaT cells were cultured in DMEM with 10% FBS, glucose, penicillin, and streptomycin at 37°C with 5% CO_2_. Cell viability was assessed using an MTT assay. HaCaT cells (5 × 10^4^/well) were treated with PMLEO or PMBLEO (12.5–200 µg/mL) for 24 h. After 24 h incubation, culture medium was replaced with phosphate buffer saline (PBS) and cells were exposed to 50 mJ/cm^2^ UVB (Stratalinker 2400 equipped with 312-nm UVB bulbs, Stratagene, La Jolla, CA). Following UVB exposure, fresh DMEM was substituted for PBS, and incubation was continued for 24 h. Formazan crystals were dissolved in dimethyl sulfoxide (DMSO), and absorbance was measured at 570 nm using an ELISA microplate reader.

### Assessment of intracellular production of ROS

Intracellular ROS levels in HaCaT cells were measured using DCFH_2_-DA fluorescence as described previously [22]. Cells (2 × 10⁵/dish) were pretreated with PMLEO (25–100 µg/mL) or resveratrol (40 µM) for 2 h, exposed to 50 mJ/cm^2^ UVB, then incubated for 1 h. After PBS washing, cells were treated with 10 µM DCFH_2_-DA in DMEM for 30 min. Fluorescence intensity was measured (excitation/emission: 485/535 nm) using a Hidex Oy spectrophotometer (Hidex Oy, Turku, Finland). ROS production was expressed as a percentage relative to control cells.

### Preparation and analysis of protein extracts from HaCaT Cells

HaCaT cells (1 × 10⁶/dish) were pretreated with PMLEO (25–100 µg/mL) or resveratrol (40 µM) for 2 h, then exposed to 50 mJ/cm^2^ UVB for 30 min to 24 h. Protein extraction was performed using radioimmunoprecipitation assay (RIPA) buffer, and concentrations were measured via the Bradford assay. Proteins (100 µg/sample) were resolved on SDS-PAGE, transferred to PVDF membranes, and blocked with 5% milk. After incubation with primary and HRP-conjugated secondary antibodies, proteins were detected using ECL reagents and imaged with ChemiDoc XRS+. Densitometric analysis was conducted using Image Lab software (Bio-Rad) as described previously [22].

### RNA extraction and quantitative PCR analysis

Total RNA was extracted using the GeneMark Total RNA Purification Kit (GeneMark, New Taipei City, Taiwan). cDNA was synthesized from 2 µg RNA using the SuperScript IV First-Strand Synthesis Kit (Invitrogen, Waltham, MA). qPCR was performed with Power SYBR Green Master Mix on an Applied Biosystems Real-Time PCR System (Applied Biosystems, Foster City, CA). The protocol included denaturation at 96°C, annealing at 50°C, and extension at 72°C for 40 cycles. HO-1 expression was normalized to GAPDH, and relative mRNA levels were quantified using the 2^Δ*Ct*^ method. Primer sequences were: HO-1 (Forward: 5′-TCAACGGCACAGTCAAGG-3′, Reverse: 5′-ACTCCACGACATACTCAGC-3′) and GAPDH (Forward: 5′-GATCATCAGCAATGCCTCCT-3′, Reverse: 5′-TTCCTCTTGTGCTCTTGCTG-3′).

### Immunofluorescence analysis

HaCaT cells (2 × 10^4^/well) were cultured in 8-well chamber slides and pre-treated with PMLEO (100 µg/mL) or resveratrol (40 µM) for 2 h. After UVB exposure (50 mJ/cm^2^, 1 h), cells were fixed with 4% paraformaldehyde, permeabilized with 0.1% Triton X-100, and blocked with 10% FBS. They were incubated with primary antibodies (Nrf2, phospho-p53) for 2 h, followed by fluorescein isothiocyanate (FITC)-conjugated secondary antibodies (1 h) and DAPI staining (5 min). Fluorescence images were captured at 40× magnification using a Motic fluorescence microscope.

### ARE promoter activity

A luciferase reporter assay (Promega) was used to assess ARE promoter activity. HaCaT cells (5 × 10^4^/well) were plated in 24-well plates and cultured in serum-free DMEM for 5 h. Cells were transfected with ARE or β-galactosidase plasmids using Lipofectamine 2000. After transfection, they were pre-treated with PMLEO (12.5–100 µg/mL) or resveratrol (40 µM) for 2 h, exposed to UVB (50 mJ/cm^2^), and incubated for 1 h. Luciferase activity was measured using a luminometer (Hidex Oy) and normalized to β-galactosidase. Data represent the mean of three independent experiments.

### Nrf2 silencing by siRNA

Nrf2 silencing was achieved using siRNA transfected into cells with Lipofectamine RNAiMax (Invitrogen) following the manufacturer's protocol. HaCaT cells were seeded in 6-well plates to reach 40–60% confluence at transfection. The next day, cells were transfected in Opti-MEM (Invitrogen) by mixing 100 pM of siRNA with RNAiMax reagent. After a 25-min incubation at room temperature, the siRNA/RNAiMax complex was added to cells, making up a final volume of 1 mL. After a 6-h incubation, the medium was substituted with 2 mL of growth media, and the cells were grown at 37°C. Prior to UVB exposure, cells were pre-treated with PMLEO (100 µg/mL) or resveratrol (40 µM) for 2 h. Cells were then irradiated with 50 mJ/cm^2^ UVB and further incubated for 1–24 h. Subsequently, cells were lysed for ROS assay.

### Quantification of intercellular α-MSH

A human α-MSH ELISA reagent from Elabscience Biotechnology Inc. (Hubei, China) was employed to ascertain the concentration of α-MSH in culture media. Cells were seeded in 6 cm dishes at a density of 2 × 10^5^ cells per dish and pre-incubated with PMLEO (25–100 µg/mL) or resveratrol (40 µM) for 2 h. Cells were then irradiated with 50 mJ/cm^2^ UVB and subsequently incubated for an additional 24 h. In accordance with the supplier's protocol, immunoassay was performed on the culture media. Immunoreactivity was assessed at a wavelength of 450 nm with the help of a microplate reader, with α-MSH concentrations established based on a standard curve supplied by the kit.

### Statistical analysis

The results are presented as mean ± standard deviation (SD). Statistical analyses were performed using GraphPad Prism version 10 (GraphPad Software, San Diego, CA, USA). Differences between groups were assessed using one-way analysis of variance (ANOVA) followed by Dunnett's post hoc test for multiple comparisons. A *p*-value of less than 0.05 was considered statistically significant when comparing control groups to UVB-treated groups. For comparisons between UVB + sample-treated groups and UVB-alone groups, significance levels were indicated as follows: *p < 0.05, **p < 0.01, ***p < 0.001, and ****p < 0.0001.

## Results

### Cytotoxicity of *P. morrisonicola* essential oils on HaCaT cells

**Figure 1.**
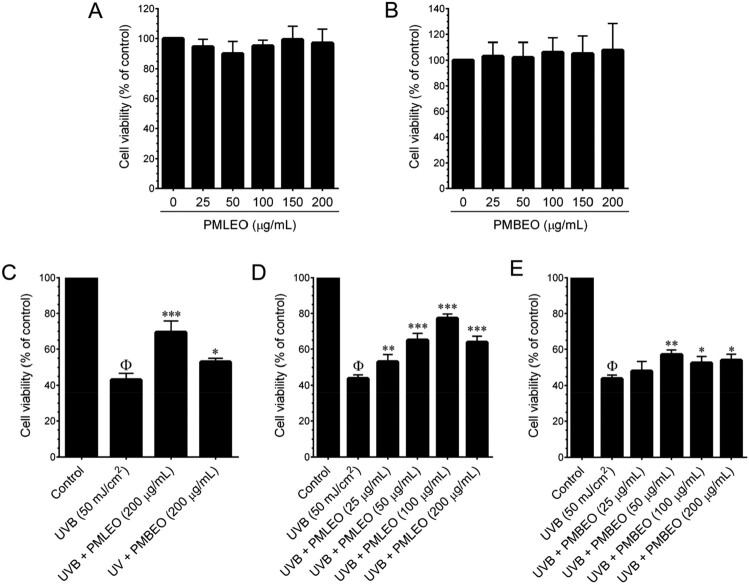
Cytotoxic effect of PMLEO, PMBEO, and UVB on HaCaT cells. (A) HaCaT cells were incubated with increasing concentrations of PMLEO (25–200 µg/mL) for 24 h. (B) HaCaT cells were incubated with increasing concentrations of PMBEO (25–200 µg/mL) for 24 h. (C) HaCaT cells were pre-treated with indicated concentrations of PMLEO and PMBEO for 2 h and then exposed to UVB (50 mJ/cm^2^) for 24 h. (D) HaCaT cells were pre-treated with various concentrations of PMLEO (25–100 µg/mL) for 2 h and then exposed to UVB (50 mJ/cm^2^) for 24 h. (E) HaCaT cells were pre-treated with various concentrations of PMBEO (25–100 µg/mL) for 2 h and then exposed to UVB (50 mJ/cm^2^) for 24 h. The cell viability was measured by MTT assay. The percentage of cell viability was compared with the control (0.01% DMSO) group. Values represent the mean ± SD of three independent experiments. Statistical significance was set at ^Φ^*P* < 0.05 compared to control vs. UVB (50 mJ/cm^2^) and *P < 0.05, **P < 0.01, ***P < 0.001 compared with UVB alone treatment group *vs*. UVB + sample treatment groups.

Cytotoxicity of PMLEO and PMBLEO was assessed in HaCaT cells by treating them with varying concentrations (12.5–200 µg/mL) for 24 h, with no significant effect on viability up to 200 µg/mL (Figure 1(A,B)). Non-cytotoxic concentrations (100 µg/mL) were selected for further experiments. UVB irradiation (50 mJ/cm^2^) reduced cell viability, but pre-treatment with PMLEO and PMBLEO (100 µg/mL) significantly protected against UVB-induced cell death. PMLEO showed superior protection compared to PMBLEO (Figure 1(C)), with effects being dose-dependent. Molecular mechanisms of PMLEO-mediated protection were further explored (Figure 1(D,E)).

### PMLEO mitigates UVB-induced oxidative stress in HaCaT cells by stimulating endogenous antioxidants

**Figure 2.**
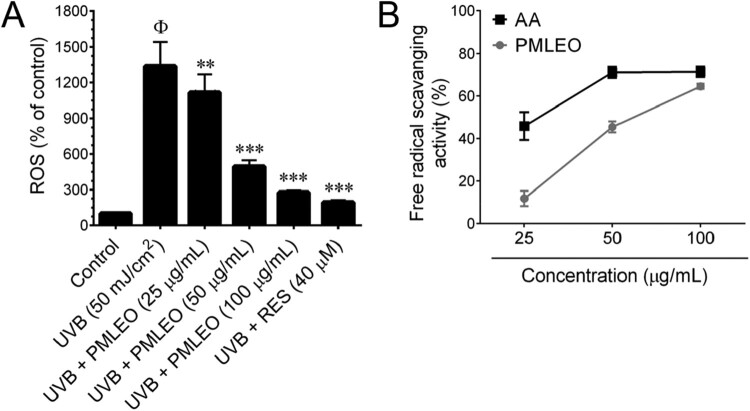
PMLEO inhibits UVB-induced oxidative stress in HaCaT cells. (A) PMLEO inhibits UVB-induced intracellular ROS generation in HaCaT cells. (B) The free-radical scavenging activity of PMLEO was determined by DPPH assay. Ascorbic acid (AA) was used as positive control. Values represent the mean ± SD of three independent experiments. Statistical significance was set at ^Φ^*P* < 0.05 compared to control *vs*. UVB (50 mJ/cm^2^) and **P < 0.01, ***P < 0.001 compared with UVB alone *vs*. UVB + sample treatment groups.

**Figure 3.**
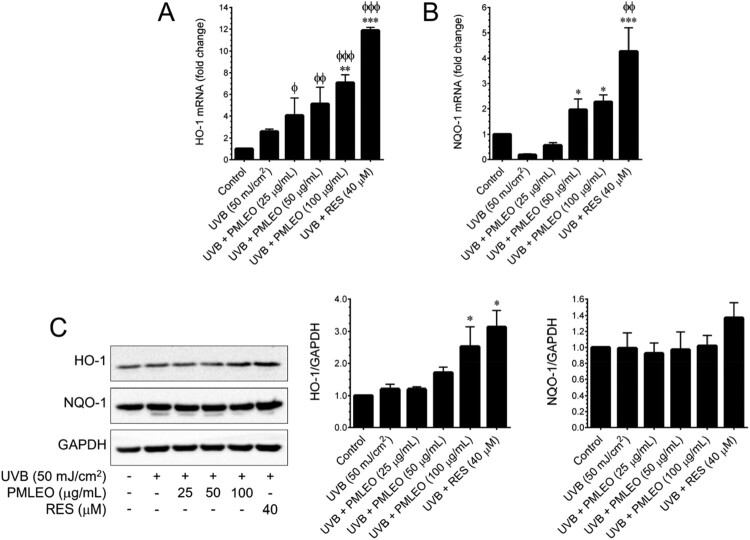
PMLEO prevents UVB-induced oxidative stress in HaCaT cells by employing endogenous anti-oxidants. (A,B) To quantify the mRNA expression level of *HO-1* and *NQO-1* HaCaT cells were pre-incubated with PMLEO (25–100 µM) or resveratrol (40 µM) and then exposed to UVB (50 mJ/cm^2^) for 6 h. Total RNA was extracted and subjected to Q-PCR analysis. Relative *HO-1* and *NQO-1* mRNA level were normalized with GAPDH mRNA. (C) To determine the protein expression levels of HO-1, NQO-1, and γ-GCLC, HaCaT cells were pre-incubated with PMLEO (25–100 µM) for 24 h. Total cell lysates were prepared and subjected to western blot analysis. Histogram shows the relative protein expression levels of HO-1, NQO-1, and γ-GCLC, which are normalized with an internal control GAPDH. Values represent the mean ± SD of three independent experiments. Statistical significance was set at ^ϕ^P < 0.05, ^ϕϕ^P < 0.01, ^ϕϕϕ^P < 0.001 compared with control a *vs*. sample treatment groups. *P < 0.05, **P < 0.01, ***P < 0.001 compared with UVB alone *vs*. UVB + sample treatment groups.

Following exposure to UVB (50 mJ/cm^2^), intracellular ROS levels in HaCaT cells were quantified using DCFH_2_-DA fluorescence spectrophotometry. UVB irradiation significantly increased intracellular ROS content by 7.3-fold, whereas pre-treatment with PMLEO dose-dependently reduced ROS levels in UVB-exposed cells (Figure 2(A)). A DPPH free-radical scavenging test was performed to evaluate the direct free-radical scavenging capabilities of varying doses of PMLEO and ascorbic acid (vitamin C). PMLEO exhibited modest free-radical scavenging effects with an IC50 value of 73.97 µg/mL, while ascorbic acid showed stronger activity with an IC_50_ of 27.01 µg/mL (Figure 2(B)). These findings suggest that PMLEO may activate endogenous antioxidant systems, enhancing its effectiveness against UVB-mediated oxidative stress. Additionally, we evaluated the impact of PMLEO on endogenous antioxidants including HO-1 and NQO-1 after UVB exposure. UVB treatment induced a significant increase in HO-1 and NQO-1 mRNA levels, with PMLEO pre-treatment notably enhancing HO-1 mRNA expression compared to UVB alone (Figure 3(A,B)). Western blotting revealed a trend of UVB-induced reduction in HO-1 and NQO-1 protein levels, which PMLEO pre-treatment mitigated significantly (Figure 3(C)).

### PMLEO enhances antioxidant levels through the Nrf2 pathway

**Figure 4.**
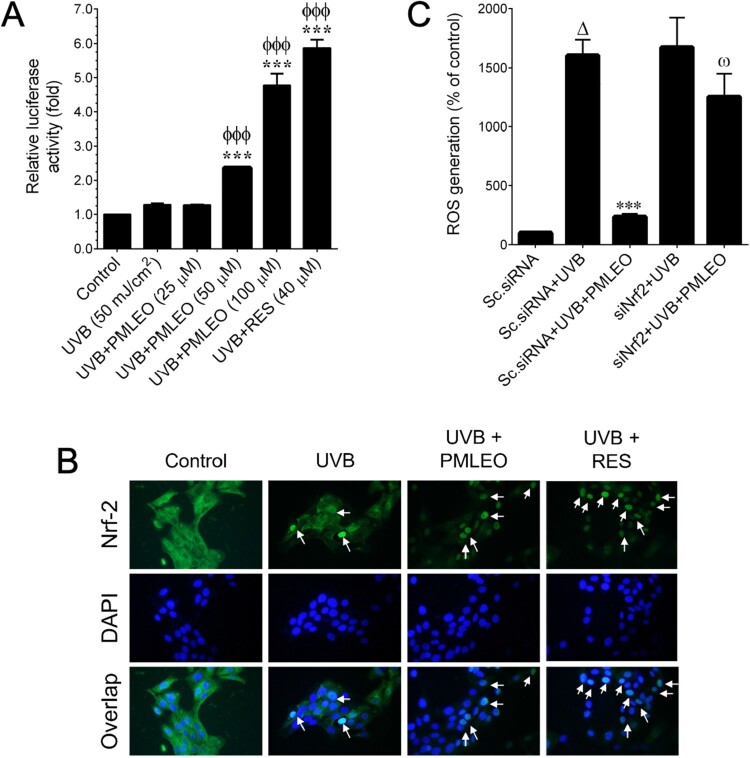
PMLEO up-regulates antioxidants *via* Nrf2 pathway. (A) To determine the Nrf2 transcriptional activity, HaCaT cells were transiently transfected with ARE promoter construct using lipofectamine and pre-incubated with PMLEO (25–100 µM) or resveratrol (40 µM) for 2 h and then exposed to UVB (50 mJ/cm^2^) for 6 h. Cell lysates were mixed with luciferase reagents and quantified using an illuminometer. Relative ARE promoter activity was calculated by dividing treated cells' relative luciferace unit (RLU) by RLU of untreated cells (control). (B) To determine the nuclear localization of Nrf2, HaCaT cells were pre-incubated with PMLEO (100 µg/mL) or resveratrol (40 µM) and then exposed to UVB (50 mJ/cm^2^) for 2 h. The protein expression and localization of Nrf2 was measured by immunofluorescence using Nrf2 specific primary antibody with FITC-conjugated secondary antibody (green). DAPI (1 µM) was used to stain the nucleus. (C) HaCaT cells were transfected with specific siRNA against Nrf2 or control siRNA. After transfection for 24 h, cells were pre-incubated with PMLEO (100 µM) for 2 h and then exposed to UVB (50 mJ/cm^2^) for 1 h. Intracellular ROS was measured by DCFH_2_-DA assay. Values represent the mean ± SD of three independent experiments. Statistical significance was set at ^ϕ^P < 0.05, ^ϕϕ^P < 0.01, ^ϕϕϕ^P < 0.001 compared with control a *vs*. sample treatment groups and **P < 0.01, ***P < 0.001 compared with UVB alone *vs*. UVB + sample treatment groups. ^Δ^*P* < 0.05 compared to control siRNA + UVB *vs*. siNrf2 + UVB. ^ω^P < 0.05 compared with siNrf2 + UVB *vs*. siNrf2 + UVB + PMLEO treatment group.

Nrf2, a redox-sensitive transcription factor, regulates ARE-dependent antioxidant genes such as HO-1, NQO-1, and γ-GCLC. To assess Nrf2 involvement in PMLEO-mediated antioxidant induction, luciferase reporter assays were performed using an ARE reporter construct. While UVB exposure showed a modest increase in luciferase activity, PMLEO pre-treatment resulted in a more than 5-fold increase, comparable to resveratrol's activity (Figure 4(A)). Immunofluorescence analysis indicated moderate Nrf2 nuclear translocation with UVB exposure, significantly enhanced by PMLEO pre-treatment (Figure 4(B)). Further validating Nrf2's role, Nrf2-specific siRNA was used to knock down Nrf2 gene expression. UVB exposure significantly increased intracellular ROS levels in scrambled siRNA-transfected cells, which PMLEO pre-treatment effectively attenuated. In siNrf2-transfected cells, UVB exposure induced higher ROS levels, partially mitigated by PMLEO (Figure 4(C)). These results underscore PMLEO's ability to reduce UVB-induced ROS production via Nrf2-dependent antioxidant pathways.

#### PMLEO modulates UVB-induced α-MSH secretion in HaCaT Cells

**Figure 5.**
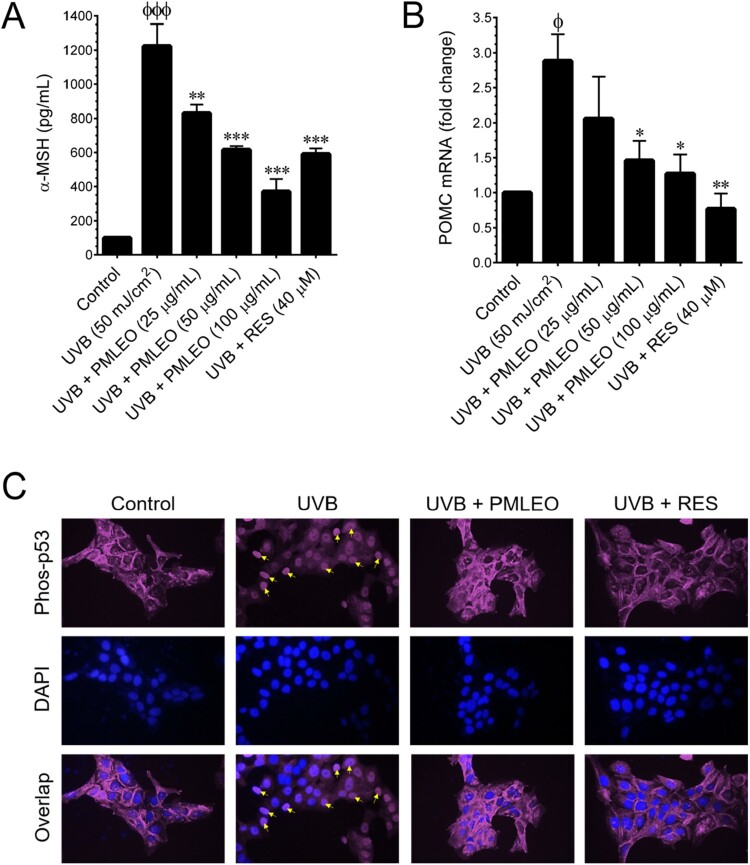
PMLEO suppresses UVB-induced α-MSH secretion by inhibiting p53 activation in HaCaT cells. (A) The intracellular levels of α-MSH were measured using a commercially available EIA kit. (B) To assess the mRNA expression of POMC, HaCaT cells were pre-treated with PMLEO (25–100 µM) or RES (40 µM), then exposed to UVB (50 mJ/cm^2^), and incubated for 6 h. Total RNA was extracted and analyzed *via* q-PCR, with POMC mRNA levels normalized to GAPDH mRNA. (C) The protein expression and localization of phosphorylated p53 (phos-p53) were evaluated by immunofluorescence using a phos-p53 specific primary antibody and a FITC-conjugated secondary antibody (purple). DAPI staining was used for nuclear visualization. Data are presented as mean ± SD from three independent experiments. Statistical significance was defined as ^ϕ^*P* < 0.05 and ^ϕϕϕ^*P* < .001for control versus UVB (50 mJ/cm^2^) and *P < 0.05, ***P < 0.01, ***P < 0.001 for UVB alone versus UVB with sample treatment. HaCaT refers to human skin keratinocytes, POMC to proopiomelanocortin, q-PCR to quantitative PCR, UVB to ultraviolet B, and α-MSH to α-melanocyte-stimulating hormone.

Upon UVB radiation, keratinocytes produce POMC through the ROS/p53 pathway, resulting in α-MSH synthesis. This α-MSH then binds to the melanocortin-1 receptor (MC1R) in melanocytes, triggering melanogenesis through cAMP/PKA-dependent CREB/MITF pathways. To assess PMLEO's impact on UVB-mediated α-MSH synthesis in skin keratinocytes, HaCaT cells were pre-incubated with PMLEO (25–100 µg/mL) for 2 h followed by UVB exposure (50 mJ/cm^2^) for 24 h. α-MSH levels were quantified using a commercial EIA kit. UVB treatment significantly increased α-MSH secretion to 1256 pg/mL over 24 h compared to non-UVB-treated cells (112 pg/mL) (Figure 5(A)). PMLEO pretreatment dose-dependently inhibited this increase, reducing α-MSH secretion to 849, 676, and 430 pg/mL at 25, 50, and 100 µg/mL, respectively. Notably, PMLEO at 100 µg/mL showed remarkable suppression comparable to 40 µM resveratrol (570 pg/mL), a positive control drug. Previous studies have indicated that UVB exposure induces POMC mRNA expression in skin keratinocytes. Consistent with this, our qPCR analysis confirmed UVB-induced upregulation of POMC mRNA in HaCaT cells. PMLEO pretreatment attenuated UVB-induced POMC at concentration-dependent manner (Figure 5(B)), suggesting that PMLEO's inhibition of α-MSH secretion may involve suppression of POMC induction in HaCaT cells.

### PMLEO regulates the activation of p53 triggered by UVB exposure in HaCaT cells

p53 plays a crucial role in regulating POMC expression in response to UVB exposure, with p38 MAPK being a key mediator of p53 phosphorylation at serine 389. We investigated whether PMLEO affects UVB-mediated transcriptional activity of p53 and nuclear export. Immunofluorescence analysis revealed that increased p53 phosphorylation and nuclear translocation following UVB exposure are signs of p53 activation (Figure 5(C)). Conversely, pre-treatment with PMLEO notably reduced the phosphorylation of p53 and its subsequent translocation to the nucleus in response to UVB exposure, suggesting a regulatory role of PMLEO in modulating p53 activity in response to UVB stress in HaCaT cells.

## Discussion

Ultraviolet radiation (UVR) is a major contributor to skin disorders, including photoaging, immune suppression, inflammation, and skin cancers. Central to UVR-induced damage is oxidative stress, where excessive reactive oxygen species (ROS) overwhelm the skin's antioxidant defenses [23]. Dietary phytochemicals, known for their low toxicity and sun-protective properties, offer a promising strategy to enhance antioxidant defenses and mitigate UVR damage. Plant-derived compounds like polyphenols, flavonoids, and carotenoids from green tea, grape seed, and tomato extracts have demonstrated significant antioxidant and anti-inflammatory effects [24]. For instance, resveratrol from grape skins activates the Nrf2/ARE pathway, a key cellular defense mechanism, protecting keratinocytes from UVR-induced damage [25]. Similarly, epigallocatechin gallate (EGCG) in green tea reduces UVB-induced DNA damage, a critical factor in skin aging and cancer [26]. These findings highlight the therapeutic potential of plant-based compounds in preventing UVR-related skin disorders, though further research is needed to optimize their bioavailability and clinical efficacy.

Essential oils, though less studied, also show promise in preventing UV-induced photodamage. Oils like rosemary, five-flavor berry, and citrus have been shown to mitigate oxidative stress by scavenging free radicals and upregulating antioxidant defenses, such as the Nrf2 pathway [27]. Terpenes, naturally occurring compounds in essential oils, act as ROS scavengers, reducing collagen degradation and inflammation caused by UVB exposure. For example, α-pinene has been shown to regulate oxidative stress and DNA damage in keratinocytes, while limonene enhances cellular antioxidant mechanisms [28]. Encapsulation of essential oils in delivery systems like cyclodextrins improves their stability, skin penetration, and bioavailability, making them viable candidates for natural photoprotection in skincare products [29].

Despite the antioxidant potential of *Pinus morrisonicola* leaf essential oil (PMLEO), its effects on UVB-induced skin damage remained unexplored. The use of PMLEO in the context of skin pathologies, particularly UV-mediated skin cancer, represents a novel area of investigation. Although direct studies on its effects in UV-induced skin cancer models are currently limited, the selection of *P. morrisonicola* in this study is grounded in its rich phytochemical profile and documented bioactivities. Notably, its essential oil contains a variety of bioactive terpenoids, including α-pinene, β-caryophyllene, and D-limonene – compounds known for their antioxidant, anti-inflammatory, and anticancer properties. These constituents are particularly effective in neutralizing free radicals and mitigating inflammation, both of which are critical factors in UV-induced skin damage and carcinogenesis. Furthermore, while no prior studies have investigated the bioactivities of *P. morrisonicola* in animal models, related species such as *Pinus pinaster* have demonstrated photoprotective effects in UV-related skin models [30]. Together with its traditional use in Taiwanese medicine, these findings provide a strong rationale for exploring *P. morrisonicola* essential oil as a potential agent for UV-mediated skin protection.

Therefore, this study was aimed to evaluate PMLEO's protective effects on human keratinocytes exposed to 50 mJ/cm^2^ UVB radiation, which demonstrated that this dosage induces measurable oxidative stress and cellular damage in HaCaT keratinocytes while avoiding excessive cytotoxicity, thereby providing a suitable model for evaluating protective effect [22]. PMLEO, at non-toxic concentrations, reduced ROS production and cell death in UVB-exposed cells. However, the underlying mechanisms of UVB-induced cell death such as direct DNA damage or apoptosis were not assessed in this study. We recognize the importance of evaluating DNA damage directly and intend to incorporate such analyses in future investigations to further substantiate the current findings. PMLEO promoted the nuclear accumulation of Nrf2, thereby enhancing the expression of antioxidant enzymes such as HO-1 and NQO-1. Notably, PMLEO significantly and dose-dependently upregulated HO-1 at both the mRNA and protein levels. In contrast, NQO-1 expression was increased only at the mRNA level, with no corresponding elevation in protein expression. This discrepancy may be due to post-transcriptional regulatory mechanisms, including translational control, mRNA stability, or protein degradation. While elevated mRNA levels indicate transcriptional activation, the synthesis of functional proteins also depends on efficient translation and protein stability. It is therefore possible that PMLEO and RES primarily induce NQO-1 transcription without sufficiently promoting its translation or preventing degradation under the experimental conditions. siRNA-mediated knockdown of Nrf2 confirmed its role in PMLEO's protective effects. Additionally, PMLEO inhibited UVB-induced α-MSH secretion by downregulating p53-mediated POMC expression, a key pathway in melanogenesis and hyperpigmentation. By disrupting this pathway, PMLEO effectively reduced UVB-induced photodamage and melanogenesis, offering potential as a natural photoprotective agent.

Furthermore, our findings demonstrate that PMLEO exerts an anti-melanogenic effect by inhibiting UVB-induced α-MSH secretion through the downregulation of p53-mediated POMC expression. This is consistent with previous studies that have highlighted the role of natural plant extracts in modulating melanogenesis pathways. For example, essential oils from *Pinus densiflora* and *Pinus pinaster* have shown inhibitory effects on melanin synthesis in UV-induced melanocytes, often through mechanisms involving suppression of MITF or tyrosinase activity [31, 32]. Moreover, studies have reported that several plant-derived compounds, such as resveratrol and epigallocatechin gallate (EGCG), can modulate p53 activity and reduce POMC expression, ultimately leading to decreased α-MSH levels and melanin production [33, 34]. These findings suggest that targeting the p53-POMC-α-MSH axis may be a common and effective strategy for natural anti-melanogenic agents. The novel observation in our study extends this understanding to *P. morrisonicola*, a species whose bioactivity has not been previously explored in this context. This not only broadens the spectrum of anti-melanogenic botanicals but also highlights the potential of PMLEO as a candidate for cosmetic or therapeutic applications against hyperpigmentation.

## Conclusion

In conclusion, this study provides experimental evidence that essential oils extracted from Pinus morrisonicola leaves protect skin keratinocytes from photodamage caused by UVB through reducing intracellular ROS levels and preventing cell death. Mechanistically, PMLEO up-regulates Nrf2-associated cellular antioxidants, particularly HO-1 and NQO-1, crucial for skin protection against UVB-induced oxidative stress. Additionally, PMLEO inhibits UVB-mediated α-MSH secretion by downregulating p53-mediated POMC expression, suggesting its potential to prevent UVB-induced photodamage and melanogenesis. These findings suggest that PMLEO could serve as a promising intervention for UVB-induced photodamage, particularly beneficial for individuals exposed to daily UVB radiation, such as outdoor workers. PMLEO holds potential as a novel dermato-protective agent for cosmetic applications, supporting skin integrity and barrier function.

## Data Availability

The data that support the findings of this study are available from the corresponding author upon reasonable request.
